# Association Between Hepatic Steatosis and Deterioration of Metabolic Health in Obese Individuals: A 12‐Year Follow‐Up of the Tehran Lipid and Glucose Study

**DOI:** 10.1002/edm2.70156

**Published:** 2026-02-09

**Authors:** Behnaz Abiri, Mohammad Nikoohemmat, Maryam Mahdavi, Amirhossein Ramezani Ahmadi, Majid Valizadeh, Fereidoun Azizi, Farhad Hosseinpanah

**Affiliations:** ^1^ Obesity Research Center, Research Institute for Metabolic and Obesity Disorders, Research Institute for Endocrine Sciences Shahid Beheshti University of Medical Sciences Tehran Iran; ^2^ Isfahan Endocrine and Metabolism Research Center Isfahan University of Medical Sciences Isfahan Iran; ^3^ Endocrine Research Center, Research Institute for Endocrine Sciences Shahid Beheshti University of Medical Science Tehran Iran

**Keywords:** fatty liver index, hepatic steatosis, metabolically healthy obesity, metabolically unhealthy obesity, transition of obesity phenotype

## Abstract

**Background:**

Uncertainty surrounds the relationship between metabolic decline and fatty liver disease. The purpose of this study was to evaluate the effect of hepatic steatosis (HS) on the transition from metabolically healthy obesity (MHO) to metabolically unhealthy obesity (MUO) and to determine whether the Fatty Liver Index (FLI) can predict this progression.

**Methods:**

In this prospective cohort research, participants in the Tehran Lipid and Glucose Study (TLGS) comprised 593 MHO adults who were at least 40 years old at baseline. The subjects were followed up for a period of 12 years to see if the subjects' metabolically healthy obesity phenotype changed to an unhealthy one. The body mass index (BMI) of 30.0 kg/m^2^ was used to characterise obesity. The concept of metabolically healthy (less than two variables) or metabolically abnormal (two or more) was based on four metabolic parameters: low high density lipoprotein‐cholesterol (HDL‐c) concentration, hypertension, hypertriglyceridemia, and impaired fasting glucose. The technique used to construct the fatty liver index (FLI), which serves as an indication of HS, was based on waist circumference (WC), triglycerides, BMI, and gamma‐glutamyl transferase.

**Results:**

During a median of 4.81 years of follow‐up (interquartile range 1.75–10.74 years), 72.2% (*n* = 428) of MHO individuals transitioned to the MUO phenotype. Transitioning participants exhibited higher FLI scores, BMI, waist circumference, and unfavourable metabolic profiles compared to non‐transitioning participants. Cox regression analysis revealed that hepatic steatosis (HR: 1.369; 95% CI: 1.014–1.848), lower physical activity (HR: 1.267; 95% CI: 1.035–1.551), and a higher TyG index (HR: 3.208; 95% CI: 1.546–6.657) were significant predictors of transition to MUO.

**Conclusion:**

Hepatic steatosis at baseline is an independent risk factor for progression from metabolically healthy status to metabolically abnormal phenotype in obese individuals.

## Graphical abstract

This 12‐year study reveals that hepatic steatosis significantly predicts the transition from metabolically healthy obesity (MHO) to an unhealthy phenotype in obese individuals, underscoring the clinical importance of early detection and intervention to prevent metabolic deterioration.

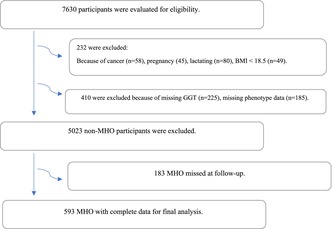

## 1 Introduction

Obesity has become a global epidemic that shares a major portion of the Global Burden of Disease [1]. The connection between obesity and metabolic irregularities, as well as chronic diseases, is well established. Metabolic disorders related to obesity, such as hyperglycemia, dyslipidemia, and hypertension, increase the risk of cardiovascular diseases (CVDs) [1]. However, the correlation between obesity and disease risk is not yet consistent [2] and over the past few decades, a subset of obese individuals has been identified without any abnormal cardiovascular profiles and normal insulin sensitivity. This phenotype has been referred to as metabolically healthy obesity (MHO) [3].

Accumulating data suggests that metabolically healthy people with obesity lack a completely benign condition [2], as around 33% of MHO subjects convert to a status of metabolically unhealthy obesity (MUO) after 5.5–10.3 years of follow‐up and roughly 50% of them following 20 years of follow‐up [4, 5, 6, 7]. Factors including greater visceral abdominal fat, higher fasting insulin levels, higher triglycerides, and lower baseline HDL cholesterol levels are related to increased transition to MUO [3], confirming that MHO subjects in these cohorts were not free of risk factors. Thus, the prognosis of metabolically healthy subjects remains uncertain.

There are currently few studies that identify clinical and metabolic factors that predict the transition from the MHO phenotype to the MUO phenotype, and the risk of incidentally developing an abnormally high metabolism in obesity is still unclear. Therefore, identifying risk factors that assist in converting patients from MHO to a metabolically abnormal phenotype is useful for clinical settings.

Among the most common chronic liver diseases worldwide is Metabolic Dysfunction‐Associated Fatty Liver Disease (MAFLD) [8]. MAFLD is characterized by fat accumulation in the liver that accounts for more than 5% of its weight [9] and can range from simple steatosis to steatohepatitis, fibrosis, and cirrhosis. This disease can raise the risk of liver‐related morbidity and mortality, as well as diabetes, chronic kidney disease, and cardiovascular disease [10].

Early detection of those who are more susceptible to fatty liver disease (FLD) may enable the implementation of preventive measures that can impede the onset and advancement of illnesses connected to the liver as well as cardiovascular disease (CVD) [9] Magnetic resonance imaging, biopsy, and hepatic ultrasonography are methods that can be used for conventional diagnosis of FLD [9].

Unfortunately, these diagnostic tools have limited applicability in general clinical practice. Being a prevalent disease in the general population, MAFLD requires a simple surrogate screening marker in clinical practice [9]. Recently, the fatty liver index (FLI), based on body mass index (BMI), waist circumference (WC), triglycerides (TG), and gamma‐glutamyl transferase (GGT) [9], has emerged as a simple and economical alternative for mass screening for hepatic steatosis with reasonable sensitivity and specificity [11]. While studies such as that by Heianza et al. have demonstrated the predictive value of fatty liver for incident type 2 diabetes in non‐obese Japanese individuals [12], data on its role specifically in the transition from metabolically healthy to unhealthy phenotypes among those with established obesity remain less explored.

The purpose of this study was to evaluate the effect of hepatic steatosis (HS) on the transition from MHO to MUO. We aimed to determine whether the FLI can serve as a clinically useful predictor for identifying individuals with MHO who are at risk of progressing to MUO. This study highlights the role of hepatic steatosis in metabolic deterioration and assesses the potential of FLI as a practical tool for early risk stratification in obese populations.

## 2 Methods

### 2.1 Study Population

The Tehran Lipid and Glucose Study (TLGS), a population‐based study on a representative sample of Tehran residents in district‐13, was the basic framework for this prospective study. The study area included 13 Km^2^ of District 13, located in the eastern region of Tehran City. The study's objectives were to determine the prevalence of risk factors for non‐communicable diseases and to develop healthy lifestyles to mitigate these risk factors. Shahid Beheshti University of Medical Sciences and health Services authorised and overlooked the study. Three medical health clinics were chosen in this area as each center possessed field data on 90% of the local families. Three‐year follow‐up investigations were conducted on the subjects, and the initial measures were recorded. Until now, seven TLGS stages have been completed. Design, reasoning, data collection methods, and sampling approach for the current study were based on previous publications [13, 14]. Initially, using multistage stratified cluster sampling, a 15,005‐person sample was selected making sure that all participants were aged ≥ 3. A total of 593 participants that had a MHO status and aged > 40 who had data on the level of GGT, anthropometric, and metabolic indices from phase III (2006–2008) were selected by random sampling method. These subjects were followed up dynamically until phase VII (2020–2022). Follow‐up durations varied among participants, and to describe the distribution of follow‐up times, the median follow‐up duration (4.8 years) and interquartile range (1.75–10.74 years) were calculated and reported.

Participants aged 40 years and older were included to focus on the population at higher cardiometabolic risk, as metabolic deterioration and transition from MHO to MUO are more prevalent in midlife and beyond. This age threshold helps control for age‐related metabolic variability and ensures relevance to clinical practice in populations most at risk.

Carefully considered inclusion and exclusion criteria were used to select participants, taking into account their health status at baseline. We thoroughly evaluated demographic data to comprehensively understand participants' general health. To ensure a robust analysis of healthy individuals at baseline, we excluded those with underlying medical conditions that could introduce confounding variables or bias the follow‐up analysis.

**FIGURE 1:**
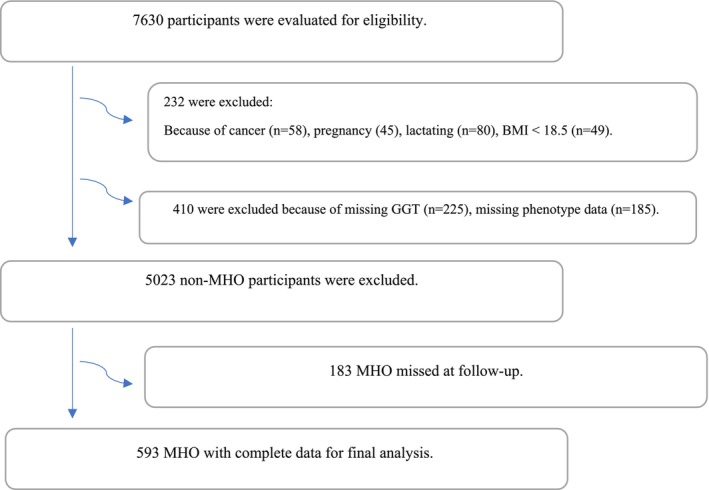
Flowchart illustrating the participant recruitment process.

Exclusion criteria consisted of: (1) Participants with conditions affecting metabolic or hepatic outcomes at baseline (including cancer, cardiovascular disease (CVD) or its history, pregnancy, or lactation); (2) Participants with missing crucial baseline data (GGT levels or metabolic phenotype); (3) Participants with a baseline BMI < 30 kg/m^2^ (i.e., non‐obese); and (4) Participants initially classified as MHO who were lost to follow‐up at the 12‐year assessment. The final longitudinal analysis comprised 593 participants with complete baseline data and follow‐up information at year 12 (Figure 1). It is important to note that our analysis focused on baseline predictors (including hepatic steatosis as assessed by FLI) and did not track changes in hepatic steatosis status or specific causes of attrition (such as mortality causes) over the 12‐year period. Future studies with continuous monitoring are warranted to address these important aspects. This study complied with the Declaration of Helsinki. Approval for this study was obtained from the Ethics Committee of the Research Institute for Endocrine Sciences (RIES) at Shahid Beheshti University of Medical Sciences (code: IR.SBMU.MSP.REC.1402.404). All participants provided written informed consent prior to participating in the study.

### 2.2 Measurements of Variables

Interviews by trained individuals were conducted in‐person utilising pre‐tested questions. First, data on age, medication use, and family history of illnesses were gathered. Using data from a modifiable activity questionnaire (MAQ), the degree of physical activity was evaluated [15]. Participants were asked to be weighed with minimal clothes and without shoes, rounding to the nearest 100 g. The subject's height was measured using a tape measure in an upright standing position with shoulders in a normal state. The Body Mass Index (BMI) was calculated as weight in kilograms divided by height in meters squared. Waist circumference was measured at the level of the umbilicus using an un‐stretched tape meter, without any pressure to the body surface, and was recorded to the nearest 0.1 cm. All measurements were taken by the same person.

A trained medical professional measured each participant's systolic and diastolic blood pressure (SBP and DBP) while the participant was seated, following a 15‐min rest period to ensure hemodynamic stabilisation. Measurements were taken using a mercury sphygmomanometer, which was the standard and most accurate device available during the initial phases of data collection. The cuff was placed on the participant's upper right arm at heart level, following standard protocols. The first measurement was used to determine the peak inflation level for cuff inflation, and two consecutive readings were obtained and averaged to calculate the participant's blood pressure. Although current guidelines recommend shorter rest periods and the use of validated automated or semi‐automated devices, our protocol reflects the best practices at the time of data collection and was consistently applied throughout the study to maintain measurement reliability.

Blood samples were collected from each participant between 7:00 and 9:00 a.m. following an overnight fast of at least 12 h, with some fasting durations extending up to 14 h due to scheduling considerations. Using glucose oxidase and enzymatic colorimetry, the fasting glucose levels were determined. The enzymatic colorimetric approach was used to measure the amounts of triglycerides (TGs) and serum total cholesterol (TC), utilizing glycerol phosphate oxidase, cholesterol esterase, and cholesterol oxidase, respectively.

After lipoproteins containing apolipoprotein B were precipitated with phosphotungstic acid, HDL‐C (high‐density lipoprotein cholesterol) was measured. Utilising commercial kits from Pars Azmoon Inc., Tehran, Iran, all biochemical assays were carried out on the day of the sample using the Vital Scientific Selectra 2 auto‐analyser located in Spankeren, The Netherlands. All samples were subjected to analyses once quality control was established. The coefficients of variation (CVs) for glucose, TC, TG, and HDL‐C were less than 2.3%, 2%, and 3%, respectively, both within and across assays.

The enzymatic colorimetric technique and kit from Delta Darman Part, Tehran, Iran were used to assess the amount of gamma‐glutamyltransferase (GGT), with intra‐ and inter‐assay CVs of less than 2.7 and 3.6, respectively.

At the RIES research facility, every measurement was done at the same time.

### 2.3 Definition of Terms

#### 2.3.1 Definition of Obesity Phenotypes

Considering BMI ≥ 30 kg/m^2^ as a cutoff is a logical approach to define the obesity phenotype [16].

According to the Joint Interim Statement (JIS) [17], abnormal metabolic components were defined as follows: (i) serum TG ≥ 150 mg/dL or taking lipid‐lowering drugs; (ii) HDL‐C < 40 mg/dL in men and < 50 mg/dL in women, or taking lipid‐lowering drugs; (iii) SBP ≥ 130 mmHg or DBP ≥ 85 mmHg, or taking antihypertensive drugs; and (iv) fasting blood glucose ≥ 100 mg/dL or receiving diabetes treatment. Subjects that portrayed less than 2 items from the JIS were defined as metabolically healthy, while participants with 2 or more components of the JIS were classified as metabolically unhealthy individuals. The subjects were then further classified into two groups: those with a BMI ≥ 30 kg/m^2^ who were metabolically healthy (MHO) and those with a BMI ≥ 30 kg/m^2^ who exhibited a metabolically unhealthy profile (MUO).

#### 2.3.2 Definition of Hepatic Steatosis

Confirmation of the HS diagnosis occurred when the score of FLI, which is calculated using a formula including WC, BMI, GGT, and TG, is equal to or more than 60 [18]. The formula is as follows:

$$\mathrm{FLI}=\frac{e^{0.953\times \ln\left(\mathrm{TG}\right)+0.139\times \mathrm{BMI}+0.718\times \ln\left(\mathrm{GGT}\right)+0.053\times \mathrm{WC}-15.745}}{1+e^{0.953\times \ln\left(\mathrm{TG}\right)+0.139\times \mathrm{BMI}+0.718\times \ln\left(\mathrm{GGT}\right)+0.053\times \mathrm{WC}-15.745}}\times 100$$

The FLI was calculated using baseline data on WC, BMI, TG, and GGT obtained at the initial study visit (phase III). These baseline FLI values were used to evaluate the predictive relationship between hepatic steatosis and subsequent transition from MHO to MUO over the follow‐up period.

#### 2.3.3 Definition of Triglyceride‐Glucose Index (TyG Index)

The TyG index is a widely used surrogate marker of insulin resistance, calculated from fasting triglyceride and glucose levels. Although it does not directly measure insulin, it has been validated against hyperinsulinemic‐euglycemic clamp and HOMA‐IR in multiple populations [19]. Its specificity for detecting insulin resistance varies across studies, and it should be interpreted as a metabolic risk marker rather than a direct measure of insulin sensitivity.

$$\mathrm{TyG}\;\text{Index}=\ln\;\left(\mathrm{TG}\;\left(\mathrm{mg}/\mathrm{dl}\right)\times \mathrm{FPG}\;\mathrm{mg}/\mathrm{dL}/2\right)$$

### 2.4 Statistical Analysis

Continuous variables were expressed as mean (standard deviation) or medians (interquartile ranges; 25th–75th percentile), and categorical variables were expressed as number (percentage). Independent *t*‐tests were used to compare means between groups for normally distributed quantitative variables, while the Mann–Whitney test was applied for non‐normally distributed quantitative variables. Categorical variables were analysed using Pearson's chi‐squared test. To assess the relationship between the presence of hepatic steatosis (HS) and the transition from the metabolically healthy obesity (MHO) phenotype to the metabolically unhealthy obesity (MUO) phenotype, hazard ratios (HRs) were estimated using Cox proportional hazards regression models. Univariate analyses were first conducted to identify potential predictors of transition. Subsequently, multivariate Cox regression analyses were performed to examine whether HS was independently associated with transition, adjusting for clinically relevant covariates and potential confounders identified based on prior literature and univariate results. To assess potential multicollinearity among predictors, variance inflation factors (VIFs) were calculated for all variables included in the final models, indicating no substantial multicollinearity that would bias the regression estimates. These covariates included age, sex, baseline BMI, waist circumference, systolic and diastolic blood pressure, total cholesterol, high‐density lipoprotein cholesterol (HDLc), physical activity level, and the TyG index. Including these variables allowed us to account for the complex interplay of metabolic and lifestyle factors impacting metabolic health. The date of incident transition from MHO to MUO was defined as the midpoint between the follow‐up visit date when MUO was first diagnosed and the most recent prior visit without MUO diagnosis. For participants who did not transition to MUO during the follow‐up period (censored observations), the follow‐up time was calculated as the interval between their baseline visit and their last recorded visit. All statistical analyses were conducted using Stata version 14.0 (Stata Corp. LLC, TX, USA). A two‐tailed P‐value of less than 0.05 was considered statistically significant.

## 3 Results

### 3.1 Participant Characteristics

At baseline, the study included 593 participants with a MHO phenotype. All characteristics presented in Table 1 refer to baseline measurements unless otherwise specified. The mean age of participants was 47.0 ± 10.4 years, with a gender distribution of 74.4% females and 25.6% males. The mean BMI and waist circumference of the total participants were 33.2 ± 3.0 kg/m^2^ and 101.0 ± 9.4 cm, respectively. There were significant differences in BMI, waist circumference, smoking status, systolic and diastolic blood pressure, arterial hypertension, fasting glucose, total cholesterol, HDL‐c, physical activity, hepatic steatosis, and FLI score between males and females (*p* < 0.05).

**TABLE 1:** General characteristics of study subjects at baseline and after 12 years follow‐up.

Variables	Baseline characteristics				After 12 years follow‐up							
					Without transition				With transition			
	Total *N* = 593	Males *N* = 152	Females *N* = 441	*p*	Total *N* = 165	Males *N* = 43	Females *N* = 122	*p*	Total *N* = 428	Males *N* = 109	Females *N* = 319	*p*
Age^a^, years	47.0 (10.4)	45.6 (11.5)	47.4 (10.0)	0.066	55.9 (11.1)	54.6 (12.2)	56.4 (10.7)	0.378	53.8 (10.3)	52.2 (11.4)	54.4 (9.8)	0.054
Sex^b^ (%)	100	25.6	74.4	—	100	26.1	73.9	—	100	25.5	74.5	—
BMI^a^, kg/m^2^	33.2 (3.0)	32.4 (2.5)	33.5 (3.1)	< 0.001	33.4 (4.1)	32.8 (4.5)	33.6 (4.0)	0.251	34.4 (3.9)	33.1 (3.3)	34.9 (4.0)	< 0.001
Family history of diabetes^c^, *N* (%)	123 (20.9)	36 (23.8)	87 (19.9)	0.306	16 (9.8)	4 (9.3)	12 (9.9)	> 0.999	47 (11.1)	9 (8.4)	38 (12.1)	0.375
Waist circumference^a^, cm	101.0 (9.4)	107.2 (6.9)	98.9 (9.2)	< 0.001	103.8 (10.9)	109.9 (11.1)	101.6 (10.0)	< 0.001	107.1 (9.6)	109.0 (7.5)	106.4 (10.2)	0.006
Smoking^c^, *N* (%)	31 (5.2)	22 (14.5)	9 (2.0)	< 0.001	11 (6.7)	8 (18.6)	3 (2.5)	0.001	16 (3.7)	11 (10.2)	5 (1.6)	< 0.001
Systolic blood pressure^a^, mmHg	112.3 (14.8)	117.5 (16.4)	110.5 (13.7)	< 0.001	111.3 (20.6)	117.3 (15.8)	109.2 (21.7)	0.026	122.5 (16.6)	126.8 (16.9)	121.0 (16.3)	0.002
Diastolic blood pressure^a^, mmHg	74.4 (8.8)	77.1 (8.8)	73.4 (8.6)	< 0.001	74.0 (11.6)	79.0 (8.8)	72.3 (12.0)	0.001	80.7 (10.7)	85.5 (10.5)	79.1 (10.2)	< 0.001
Arterial hypertension^c^, *N* (%)	39 (6.6)	16 (10.5)	23 (5.2)	0.023	24 (14.5)	8 (18.6)	16 (13.1)	0.380	174 (40.7)	48 (44.0)	126 (39.5)	0.405
Fasting glucose^a^, mg/dL	89.4 (12.8)	91.5 (13.2)	88.7 (12.6)	0.020	92.3 (16.8)	98.8 (28.6)	90.0 (8.9)	0.054	102.0 (23.8)	105.1 (23.1)	100.9 (23.9)	0.107
Diabetes mellitus^c^, *N* (%)	12 (2.0)	4 (2.7)	8 (1.8)	0.514	5 (3.1)	4 (9.5)	1 (0.8)	0.017	46 (11.2)	15 (14.6)	31 (10.1)	0.211
Total cholesterol^a^ (mg/dL)	193.4 (35.5)	187.4 (32.5)	195.4 (36.2)	0.016	191.6 (28.6)	176 (23.7)	197.2 (28.2)	< 0.001	195.6 (35.9)	192.0 (35.5)	196.8 (36.0)	0.234
HDLc^a^, mg/dL	46.6 (10.6)	41.4 (8.3)	48.3 (10.7)	< 0.001	56.0 (10.5)	48.1 (7.9)	58.8 (9.8)	< 0.001	50.1 (11.7)	45.4 (10.1)	51.8 (11.8)	< 0.001
LDLc^a^, mg/dL	123.3 (29.7)	123.0 (28.0)	123.5 (30.3)	0.867	116.4 (24.4)	108.8 (21.0)	119.0 (25.0)	0.019	118.0 (31.8)	119.1 (28.2)	117.7 (33.0)	0.681
Triglycerides^b^, mg/dL	112 (91–136)	113.5 (91.3–136)	112 (91–136.5)	0.900	93 (76–113)	91 (76–114)	93.5 (76–112.3)	0.930	130 (95–168.8)	128 (89.5–168‐5)	131 (97–169)	0.548*
Physical activity^c^ (600 min/week)	192 (32.5)	73 (48.7)	119 (27.0)	< 0.001	59 (37.3)	17 (41.5)	42 (35.9)	0.526	158 (37.8)	57 (55.3)	101 (32.1)	< 0.001
TyG^a^	8.50 ± 0.33	8.53 ± 0.33	8.49 ± 0.33	0.182	8.35 ± 0.33	8.42 ± 0.34	8.33 ± 0.32	0.117	8.76 ± 0.43	8.78 ± 0.47	8.75 ± 0.42	0.593
GGT^a^, U/L	23.2 (21.1)	32.0 (18.9)	20.2 (20.9)	< 0.001	20.1 (12.2)	28.6 (16.9)	17.1 (8.1)	< 0.001	24.4 (23.5)	33.4 (19.5)	21.4 (24.0)	< 0.001
Hepatic steatosis^c^, *N* (%)	393 (66.3)	140 (92.1)	253 (57.4)	< 0.001	84 (50.9)	38 (88.4)	46 (37.7)	< 0.001	309 (72.2)	102 (93.6)	207 (64.9)	< 0.001
FLI^a^	66.5 (18.8)	75.9 (12.9)	63.3 (19.4)	< 0.001	59.9 (20.9)	72.7 (14.2)	55.4 (21.0)	< 0.001	69.0 (17.3)	77.1 (12.2)	66.3 (18.0)	< 0.001
Time to transition^b^, years	—	—	—	—	—	—	—	—	4.08 (1.65–7.38)	2.91 (1.61–6.79)	4.23 (1.68–7.61)	—

*Note:* Statistical tests used to calculate *p*‐values: independent t‐test for normally distributed continuous variables, Mann–Whitney U test for non‐normally distributed variables expressed as median (interquartile range), and Pearson's chi‐squared test for categorical variables.

Abbreviations: BMI, body mass index; FLI, fatty liver index; GGT, gamma‐glutamyl transferase; HDLc, high‐density lipoprotein cholesterol; LDLc, low‐density lipoprotein cholesterol; TyG, triglyceride–glucose index.

^a^ Values are expressed as mean ± standard deviation (SD).

^b^ Values are expressed as median (interquartile range; 25th–75th percentile).

^c^ Values are expressed as number (percentage).

At baseline, 66.3% of the participants had hepatic steatosis, and the mean FLI score was 66.5 ± 18.8. In Table 2, the general and health characteristics between the two groups, with and without hepatic steatosis, are compared, showing significant differences in age, BMI, waist circumference, blood pressure, glucose and lipid profiles, TyG index, GGT levels, and the number of participants who were smoking.

**TABLE 2:** Comparison of baseline characteristics of participants classified by presence or absence of hepatic steatosis at baseline.

Variable	Without steatosis (*n* = 200)	With steatosis (*n* = 393)	*p* ^†^
Age^a^, years	44.4 ± 9.2	48.3 ± 10.8	< 0.001
Sex, Female^c^, *n* (%)	188 (94)	253 (64.4)	< 0.001
BMI^a^, kg/m^2^	31.5 ± 1.3	34.0 ± 3.2	< 0.001
Waist circumference^a^, cm	92.3 ± 6.0	105.5 ± 7.5	< 0.001
Systolic blood pressure^a^, mmHg	108.4 ± 13.7	114.3 ± 14.9	< 0.001
Diastolic blood pressure^a^, mmHg	72.6 ± 9.1	75.2 ± 8.5	0.001
Arterial hypertension^c^, *n* (%)	11 (5.5)	28 (7.1)	0.450
Fasting glucose^a^, mg/dL	86.9 ± 6.8	90.7 ± 14.8	< 0.001
Diabetes mellitus^c^, *n* (%)	2 (1.0)	10 (2.6)	0.203
Total cholesterol^a^, mg/dL	186.3 ± 29.7	197.0 ± 37.6	< 0.001
HDLc^a^, mg/dL	48.0 ± 10.3	45.8 ± 10.7	0.022
LDLc^a^, mg/dL	118.4 ± 26.2	125.8 ± 31.0	0.003
Triglycerides^b^, mg/dL	95.0 (75.0–116.8)	122.0 (100.0–142.0)	< 0.001
GGT^a^, U/L	15.1 ± 4.3	27.4 ± 24.7	< 0.001
TyG index^a^	8.3 ± 0.3	8.6 ± 0.3	< 0.001
FLI^a^	44.5 ± 10.7	77.7 ± 10.2	< 0.001
Physical activity^a^, met/min	47 ± 23.5	145 ± 37.2	0.001
Smoking^c^, *n* (%)	5 (2.5)	26 (6.6)	0.033
Family history of diabetes^c^, *n* (%)	38 (19.1)	85 (21.9)	0.437

Abbreviations: BMI, body mass index; FLI, fatty liver index; GGT, gamma‐glutamyl transferase; HDLc, high‐density lipoprotein cholesterol; LDLc, low‐density lipoprotein cholesterol; TyG, triglyceride–glucose index.

^†^ *p*‐value is reported based on independent sample *t*‐test for variables with normal distribution, Mann–Whitney *U* test for variables without normal distribution, and *χ* ^2^ for categorical variables.

^a^ Values are expressed as mean ± standard deviation (SD).

^b^ Values are expressed as median (interquartile range; 25th–75th percentile).

^c^ Values are expressed as number (percentage).

### 3.2 Transitioning to MUO Phenotype

**FIGURE 2:**
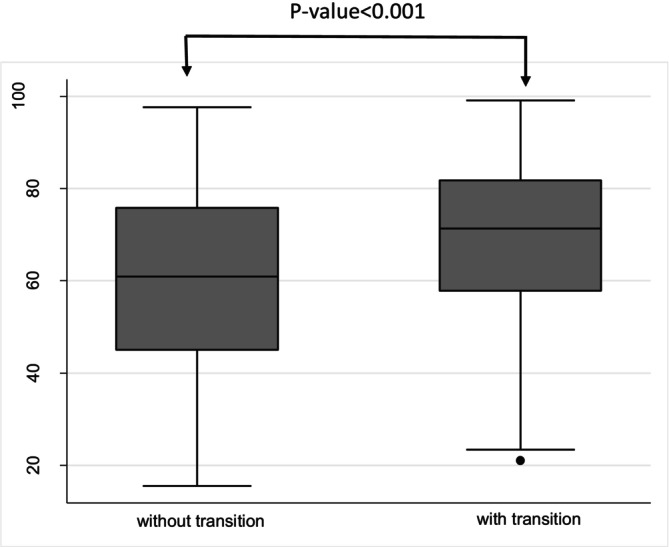
Box plot comparing Fatty Liver Index (FLI) between individuals who transitioned from metabolically healthy obesity (MHO) to metabolically unhealthy obesity (MUO) and those who did not.

Over the 12‐year follow‐up, 428 participants transitioned to the MUO phenotype, while 165 participants remained with the MHO phenotype. A higher prevalence of hepatic steatosis was observed in transitioning participants (72.2%) compared to non‐transitioning participants (50.9%). The mean FLI score was 69.0 ± 17.3 in those who transitioned and 59.9 ± 20.9 in those who did not transition after 12 years of follow‐up. Individuals who transitioned to MUO had higher FLI values compared to those who remained metabolically healthy (Figure 2).

Compared to non‐transitioning participants, transitioning participants had higher BMI (34.4 vs. 33.4 kg/m^2^), waist circumference (107.1 vs. 103.8 cm), SBP (122.5 vs. 111.3 mmHg), DBP (80.7 vs. 74.0 mmHg), arterial hypertension (40.7% vs. 14.5%), fasting glucose (102.0 vs. 92.3 mg/dL), family history of diabetes (11.1% vs. 9.8%), prevalence of diabetes (11.2% vs. 3.1%), total cholesterol (195.6 vs. 191.6 mg/dL), LDL‐C (118.0 vs. 116.4 mg/dL), triglycerides (130 vs. 93 mg/dL), and GGT (24.4 vs. 20.1 U/L). In contrast, HDL‐C levels were lower in participants who transitioned to MUO compared to those who did not transition (50.1 vs. 56.0 mg/dL).

### 3.3 Association Between HS and Transition to MUO

Table 3 presents the results of Cox regression analysis investigating the association between hepatic steatosis and the transition to MUO in univariate and multivariate models. Accordingly, the univariate models identified several significant associations, including higher risks with elevated total cholesterol, waist circumference, baseline BMI, age, triglycerides, systolic and diastolic blood pressure, lower physical activity, and the presence of hepatic steatosis. Notably, a higher TyG index was strongly associated with increased risk. In the multivariate analysis, after adjusting for other variables, higher HDLc remained protective (HR: 0.968, 95% CI: 0.957–0.979), while higher total cholesterol continued to be a risk factor (HR: 1.005, 95% CI: 1.002–1.008). Hepatic steatosis (HR: 1.369, 95% CI: 1.014–1.848) and lower physical activity (HR: 1.267, 95% CI: 1.035–1.551) remained significant, though with reduced effect sizes compared to the univariate analysis. The TyG index showed an even stronger association with risk (HR: 3.208, 95% CI: 1.546–6.657), highlighting its prominent role as a predictor. Sex (male) also emerged as a significant risk factor (HR: 1.690, 95% CI: 1.255–2.277), while other factors such as waist circumference, baseline BMI, and blood pressure measurements lost significance after adjustment.

**TABLE 3:** Associations of hepatic steatosis and metabolic factors with risk of transitioning to metabolically unhealthy obesity: Univariate and multivariate analysis.

Variable	Univariate	Multivariate
	HR (95% CI)	HR (95% CI)
HDLc, mg/dL	0.984 (0.975–0.993)	0.968 (0.957–0.979)
Total cholesterol, mg/dL	1.005 (1.003–1.008)	1.005 (1.002–1.008)
WC, cm	1.020 (1.010–1.030)	1.011 (0.994–1.028)
Baseline BMI, kg/m^2^	1.033 (1.002–1.065)	0.977 (0.935–1.021)
Age, years	1.018 (1.009–1.027)	1.009 (0.998–1.021)
Triglycerides, mg/dl	1.007 (1.004–1.009)	0.995 (0.988–1.001)
SBP, mmHg	1.012 (1.006–1.019)	1.006 (0.998–1.015)
DBP, mmHg	1.017 (1.006–1.028)	1.007 (0.993–1.021)
Sex (male: ref)	0.973 (0.783–1.210)	1.690 (1.255–2.277)
Physical activity, met/min	1.416 (1.160–1.727)	1.267 (1.035–1.551)
TyG index	2.622 (1.956–3.514)	3.208 (1.546–6.657)
Hepatic steatosis, N (%)	1.842 (1.489–2.280)	1.369 (1.014–1.848)

*Note:* Data are expressed as hazard ratios (HR) with 95% confidence intervals (CI). Hazard ratios for continuous variables are presented per 1 standard deviation (SD) increment.

Abbreviations: BMI, body mass index; CI, confidence interval; DBP, diastolic blood pressure; HDLc, high‐density lipoprotein cholesterol; LDLc, low‐density lipoprotein cholesterol; SBP, systolic blood pressure; WC, waist circumference.

Adjusted HR of 1.369 (CI: 1.014–1.848) indicates that hepatic steatosis continues to be associated with an increased risk of transitioning to MUO, though the effect is less pronounced than in the univariate analysis.

### 3.4 Association Between FLI and Transition to MUO

Table 4 shows the association between baseline FLI and the transition to MUO. The univariate model indicated that each unit increase in the baseline FLI score was associated with an increased risk of transitioning to MUO (HR = 1.015; 95% CI = 1.01, 1.02). However, in the multivariate model, this association was attenuated (HR: 1.011, 95% CI: 0.998–1.024), indicating that while FLI is a relevant predictor, its impact is less pronounced when accounting for other variables (Table 4). It should be noted that FLI includes two components that overlap with metabolic syndrome criteria—waist circumference and triglycerides—which may partially explain its predictive value. However, FLI also incorporates BMI and GGT, providing additional information related to adiposity and liver‐specific injury. Participants in higher tertiles of baseline FLI had significantly increased risks of transitioning to MUO compared to those in the lowest tertile (Tertile 1: HR = 1.878; 95% CI = 1.066, 3.309; Tertile 2: HR = 3.075; 95% CI = 1.800, 5.255; Tertile 3: reference) (data not shown). The hazard ratio for Tertile 2 is higher than Tertile 1, reflecting the non‐linear relationship between FLI levels and transition risk, likely influenced by the distribution of transition events across tertiles. This analysis highlights that a liver‐specific measure like FLI, assessed at baseline, can provide predictive value for future metabolic deterioration, even beyond the direct contribution of its overlapping metabolic components.

**TABLE 4:** Association between Fatty Liver Index (FLI) and transition to metabolically unhealthy obesity: Univariate and multivariate hazard ratios adjusted for metabolic factors.

Variable	Univariate	Multivariate
	HR (95% CI)	HR (95% CI)
HDLc, mg/dL	0.984 (0.975–0.993)	0.967 (0.956–0.978)
Total cholesterol, mg/dL	1.005 (1.003–1.008)	1.005 (1.001–1.008)
WC, cm	1.020 (1.010–1.030)	1.002 (0.981–1.023)
Baseline BMI, kg/m^2^	1.033 (1.002–1.065)	0.960 (0.914–1.008)
Age, years	1.018 (1.009–1.027)	1.012 (1.001–1.023)
Triglycerides, mg/dL	1.007 (1.004–1.009)	1.002 (0.998–1.006)
SBP, mmHg	1.012 (1.006–1.019)	1.007 (0.998–1.016)
DBP, mmHg	1.017 (1.006–1.028)	1.004 (0.990–1.018)
Sex (male: ref)	0.973 (0.783–1.210)	1.693 (1.252–2.290)
Physical activity, met/min	1.416 (1.160–1.727)	1.312 (1.073–1.605)
TyG index	2.622 (1.956–3.514)	3.055 (1.447–6.449)
FLI	1.015 (1.010–1.020)	1.011 (0.998–1.024)

*Note:* Data are expressed as hazard ratios (HR) with 95% confidence intervals (CI). Hazard ratios for continuous variables are presented per 1 standard deviation (SD) increment.

Abbreviations: BMI, body mass index; DBP, diastolic blood pressure; FLI, fatty liver index; HDLc, high‐density lipoprotein cholesterol; LDLc, low‐density lipoprotein cholesterol; SBP, systolic blood pressure; WC, waist circumference.

## 4 Discussion

In this cohort study, the MHO phenotype was found to be transient, with about 72.2% of MHO individuals progressing to the MUO phenotype over a median follow‐up period of 4.81 years. Our findings clearly demonstrate that baseline HS independently predicts the future shift from the MHO to the MUO phenotype.

Growing evidence suggests that MHO is often a transient state, with many individuals progressing to MUO over time [2, 20]. Our observed transition rate of 72.7% was higher than in some prior studies, likely reflecting differences in ethnic background, follow‐up duration, diagnostic criteria, and lifestyle factors across populations. Predictors of metabolic transition from MHO remain poorly understood. Although MAFLD is linked to insulin resistance and may signal future MUO development [2, 10, 21, 22], its predictive role in some studies diminished after adjusting for insulin resistance and other metabolic factors [10, 21, 23]. This suggests that factors beyond insulin resistance—such as systemic inflammation, oxidative stress, and gut microbiota changes—may also drive the transition to an unhealthy phenotype [2]. Emerging evidence identifies insulin resistance as the central driver of the transition from MHO to MUO [24]. It disrupts glucose and lipid metabolism, promoting hepatic fat accumulation and the development of NAFLD as a downstream manifestation. Our findings, supported by the strong predictive value of the TyG index, align with this model. Hepatic steatosis independently predicts transition but likely acts as a marker and amplifier within this insulin resistance‐driven cascade. Early identification and targeting of insulin resistance are therefore crucial to preventing adverse metabolic progression [24, 25]. It is important to note that the TyG index, while correlated with insulin resistance, is an indirect surrogate that does not incorporate insulin measurements. Therefore, our use of TyG should be interpreted as reflecting a broader metabolic dysregulation phenotype rather than specific insulin resistance per se. Future studies incorporating direct measures of insulin (e.g., HOMA‐IR, hyperinsulinemic clamp) would help clarify the distinct contributions of insulin resistance versus other metabolic disturbances in the transition from MHO to MUO.

Hepatic steatosis significantly contributes to obesity‐related metabolic dysfunction by worsening insulin resistance, promoting atherogenic dyslipidemia, and stimulating proinflammatory and vasoactive mediators [26, 27]. Bile acids, key regulators of energy balance, are often dysregulated in obesity and nonalcoholic steatohepatitis (NASH) [28]. Elevated serum bile acids correlate with insulin resistance but not with NASH‐associated necroinflammation [28]. Since NASH involves altered bile acid profiles [29], bile acids may serve as a mechanistic link between HS and the transition to MUO.

Hwang et al. reported that age, metabolic syndrome components (excluding HDL‐c), HOMA‐IR, liver enzymes, inflammation, and fatty liver predict transition from MH to MU [2]. Another study identified MAFLD as a strong predictor of metabolic transition [30]. This aligns with evidence linking visceral fat accumulation to metabolic deterioration, suggesting MAFLD—often reflecting visceral adiposity—is more metabolically significant than subcutaneous fat [2, 30].

While large studies often use liver enzymes like ALT or GGT as MAFLD proxies [31, 32], we used the FLI, a practical clinical tool based on BMI, WC, triglycerides, and GGT. FLI is a validated predictor of incident diabetes in the general and prediabetic populations [33, 34] and is recommended by EASL/EASD/EASO for large‐scale MAFLD screening [35]. A meta‐analysis of 27 studies confirmed that higher FLI scores consistently predict diabetes risk across subgroups [36]. FLI also independently predicts future cardiovascular disease risk [9]. Our results are consistent with other studies that have shown a correlation between metabolic changes in obesity and fatty liver identified via ultrasonography [2] or visceral abdominal fat assessed through computed tomography [37]. The observed non‐linear (U‐shaped) association between FLI tertiles and transition risk requires careful interpretation, as hepatic fat accumulation is generally thought to exhibit a monotonic dose–response relationship with metabolic decline. This pattern may arise from sample size constraints, variability in FLI components, or residual confounding. Analysis of individual FLI components (BMI, WC, TG, GGT) revealed largely linear relationships with metabolic deterioration, in line with known pathophysiology. Thus, the non‐linearity in the composite FLI likely reflects complex interactions among its components rather than a genuine U‐shaped risk.

In our analysis, we used baseline FLI to predict incident transition to MUO, thereby assessing its prognostic value rather than merely reflecting concurrent metabolic overlap. However, we acknowledge that FLI incorporates WC and triglycerides, which are also components of metabolic syndrome, creating partial conceptual circularity. To isolate the liver‐specific contribution, we conducted a post hoc analysis using only the liver enzyme component of FLI (GGT) as a standalone predictor. In a model adjusted for age, sex, BMI, and TyG index, elevated baseline GGT remained independently associated with transition to MUO, suggesting that liver‐specific injury, independent of general adiposity and dyslipidemia, contributes to metabolic deterioration. Future studies with direct imaging‐based hepatic fat quantification (e.g., MRI‐PDFF) or histologic assessment could further clarify the unique predictive value of liver fat per se, beyond the metabolic components shared with FLI.

We acknowledge that the FLI and TyG index share some biochemical components (e.g., triglycerides). However, these indices were chosen to represent distinct pathophysiological constructs: FLI as a surrogate for hepatic steatosis and TyG as a marker of systemic insulin resistance. In sensitivity analyses where we replaced FLI and TyG with their individual components (GGT, TG, fasting glucose), the associations between HS and transition to MUO remained significant, supporting the robustness of our findings. Nevertheless, future studies may consider using non‐overlapping biomarkers or imaging‐based fat quantification to further delineate these relationships.

Our multivariate models included covariates with potential conceptual overlap (e.g., BMI and WC; TyG index shares TG with FLI; some metabolic syndrome components define both predictors and outcome). This may have introduced multicollinearity, affecting precision and possibly attenuating the significance of FLI in tertile analyses. Future studies could use methods like factor analysis to better isolate the independent effect of hepatic steatosis. Despite this, the consistent associations in univariate and adjusted models reinforce that hepatic steatosis is a key factor in metabolic deterioration in obesity.

This study is the first prospective investigation in the MENA region to evaluate the role of hepatic steatosis, assessed via the FLI, in the transition from MHO to a metabolically unhealthy phenotype, using a relatively large and well‐characterised cohort from the TLGS. The application of a multistage stratified cluster sampling method enhances the internal representativeness of the sample. Our analysis controlled for several key metabolic and lifestyle confounders, strengthening the inference that hepatic steatosis is independently associated with metabolic deterioration in obesity. However, several limitations should be considered. First, participants were recruited from a single district of Tehran, which may limit the generalizability of the findings to the broader Iranian and other populations, despite the robust sampling method. Second, the study lacked data on body fat mass, dietary habits, and menopausal status—important factors that could influence metabolic health transitions. Third, as participants were volunteers in a health screening program, they may be more health‐conscious than the general population, potentially introducing selection bias. Fourth, due to sample size constraints, we were unable to conduct sex‐specific analyses. Fifth, hepatic steatosis was assessed only at baseline using the FLI. We lack repeated FLI measurements or imaging data at follow‐up, preventing us from analysing the progression of steatosis or its concurrent change with metabolic transition over the 12‐year period. Sixth, the use of FLI as a surrogate for hepatic steatosis introduces a degree of conceptual overlap with the metabolic syndrome criteria, as triglyceride levels are a component of both FLI and the definition of metabolically unhealthy obesity. Finally, the absence of genetic data limits our ability to account for inherited susceptibilities to hepatic steatosis and metabolic deterioration.

Future research should aim to validate these findings in larger, multicenter cohorts across diverse regions. Incorporating direct imaging‐based assessments of hepatic fat (e.g., MRI‐PDFF), repeated FLI measurements over time, genetic profiling, and detailed data on menopausal status and diet would help clarify causal pathways, minimize confounding, and improve the generalizability and clinical applicability of the results.

## 5 Conclusion

This study reveals that HS is a significant independent predictor of the transition from MHO to a metabolically unhealthy phenotype over a median follow‐up of 4.81 years. Individuals with MHO and high baseline FLI scores are at increased risk of developing metabolic abnormalities. Our findings underscore the necessity for early detection and monitoring of HS in clinical practice, challenging the perception of MHO as a benign condition. Regular screening and targeted interventions are crucial to prevent progression to more severe metabolic states. Further research is needed to better understand the relationship between hepatic steatosis and obesity metabolic phenotypes, its potential as a biomarker for phenotype transition, and the underlying mechanisms, including the role of lifestyle factors in this process.

## Author Contributions

B.A., F.H. and M.V. conceived and designed the study. B.A., M.M., M.N. and A.R.A. contributed to the interpretation of the results and wrote the first draft of the manuscript. F.H., M.V., F.A. and B.A. critically revised the manuscript. All authors have read and approved the final manuscript.

## Funding

The authors did not receive any specific funding for this study.

## Ethics Statement

This study complied with the Declaration of Helsinki and was approved by the Ethics Committee of the Research Institute for Endocrine Sciences (RIES) at Shahid Beheshti University of Medical Sciences (code IR.SBMU.MSP.REC.1402.404). All participants provided written informed consent.

## Consent

All authors have given consent for the paper to be published by the corresponding author.

## Conflicts of Interest

The authors declare no conflicts of interest.

## Data Availability

The data that support the findings of this study are available on request from the corresponding author. The data are not publicly available due to privacy or ethical restrictions.
